# RSM Based Optimization of Chemical and Enzymatic Transesterification of Palm Oil: Biodiesel Production and Assessment of Exhaust Emission Levels

**DOI:** 10.1155/2014/526105

**Published:** 2014-08-05

**Authors:** Muhammad Waseem Mumtaz, Hamid Mukhtar, Farooq Anwar, Nazamid Saari

**Affiliations:** ^1^Department of Chemistry, University of Gujrat, Pakistan; ^2^Institute of Industrial Biotechnology, GC University, Lahore 54000, Pakistan; ^3^Department of Chemistry, University of Sargodha, Sargodha 40100, Pakistan; ^4^College of Pharmacy, Salman bin Abdulaziz University, AlKharj 11942, Saudi Arabia; ^5^Faculty of Food Science and Technology, Universiti Putra Malaysia, 43400 Serdang, Selangor, Malaysia

## Abstract

Current study presents RSM based optimized production of biodiesel from palm oil using chemical and enzymatic transesterification. The emission behavior of biodiesel and its blends, namely, POB-5, POB-20, POB-40, POB-50, POB-80, and POB-100 was examined using diesel engine (equipped with tube well). Optimized palm oil fatty acid methyl esters (POFAMEs) yields were depicted to be 47.6 ± 1.5, 92.7 ± 2.5, and 95.4 ± 2.0% for chemical transesterification catalyzed by NaOH, KOH, and NaOCH_3_, respectively, whereas for enzymatic transesterification reactions catalyzed by NOVOZYME-435 and A. n. lipase optimized biodiesel yields were 94.2 ± 3.1 and 62.8 ± 2.4%, respectively. Distinct decrease in particulate matter (PM) and carbon monoxide (CO) levels was experienced in exhaust emissions from engine operating on biodiesel blends POB-5, POB-20, POB-40, POB-50, POB-80, and POB-100 comparative to conventional petroleum diesel. Percentage change in CO and PM emissions for different biodiesel blends ranged from −2.1 to −68.7% and −6.2 to −58.4%, respectively, relative to conventional diesel, whereas an irregular trend was observed for NOx emissions. Only POB-5 and POB-20 showed notable reductions, whereas all other blends (POB-40 to POB-100) showed slight increase in NOx emission levels from 2.6 to 5.5% comparative to petroleum diesel.

## 1. Introduction

At present, the world is looking for appropriate solution of energy crises as its demand is escalating day by day due to rapidly growing population and global industrialization. Currently, fossil based nonrenewable resources like petroleum, coal, natural gas, and so forth are mostly fulfilling the energy requirements of the world but their limited availability is inversely proportional to the rising energy demands. Besides, the consumption of these fossil fuels is intimidating the whole world with environmental impacts like global warming and depletion of the ozone shield [1].

Typically, the diesel engine exhaust (CO, NOx, and PM) has been categorized as carcinogenic and several studies revealed an augmented health risk like lung cancer upon long term exposure to diesel engine exhaust [2]. The main carcinogenic effects associated with diesel exhaust are attributed to soot particles inhalation as various mutagenic and carcinogenic agents (e.g., polycyclic aromatic hydrocarbons) are known to be absorbed as organic phase on particulate matter (PM) surface. PM that are readily inhaled having median dynamic diameter, that is, 0.1–0.3 *μ*m, result in deposition in alveolar region of lungs [3–6]. Researchers and environmentalists are extensively emphasizing for search of alternative and renewable fuels that can lead to cleaner environment. Although different countries throughout the world are focusing on posttreatment technologies for simultaneous PM, CO, and NOx reductions, but use of biodiesel (being renewable energy fuel) instead of diesel fuel has proved to give best results towards cleaner environment. Biodiesel has some advantages over conventional fossil diesel, especially in terms of lesser toxic emissions [7]. Previous studies revealed that the use of biodiesel instead of biodiesel or in blended form in diesel engine showed reduced levels of exhaust emissions of CO and PM, and so forth [8–10].

Optimization of biodiesel production through chemical or enzymatic transesterification process is another area of consideration. In this context, different optimization methods have been developed by the researchers with the main target of offering high yield and good quality of biodiesel product. Response surface methodology (RSM) is gaining much recognition these days as an imperative tool for the optimization of various biochemical and technological processes. Usually, RSM is considered as a combination of mathematical and statistical protocols utilized for process development, improvement, and optimization. RSM is also proved to be very helpful technique for the analysis of specific problems where a response of interest is influenced by various process variables with the objective to optimize the said response [11–13].

Although palm oil is in use as a feedstock for biodiesel production, no earlier studies are reported on the RSM based simultaneous optimization of chemical and enzymatic transesterification for production of biodiesel using palm oil. Therefore, the current study was designed with the main objective to optimize both of the chemical and enzymatic transesterification processes for palm oil biodiesel production using RSM as well as evaluation of exhaust emission levels from diesel engine operated on the synthesized biodiesel and its blends to check its compatibility as a green fuel.

## 2. Experimental Section

### 2.1. Collection of Raw Material

All the experimental work was executed using analytical/research grade chemicals and reagents. Palm oil was procured from DESCON chemicals (Pvt.) Ltd. Davis Road Lahore, Pakistan. KOH, NaOH, NaOCH_3_, methanol, toluene, isopropanol, phenolphthalein, starch, acetic acid, HCl, sodium thiosulfate, n-hexane, acetone, potassium iodide, iodine trichloride, potassium dichromate, and chloroform were obtained from Merck Chemicals, (Germany), while NOVOZYME-435 (lipase acrylic resin from Candida Antarctica) and lipase from* Aspergillus niger* were purchased from Sigma Chemical Co. (St. Louis, MO). A.n. lipase (lipase from* Aspergillus niger* immobilized on lewatite**)** was obtained from Process Biotechnology Laboratory, Department of Chemistry, GC University, Lahore, Pakistan.

### 2.2. Physicochemical Characteristics of Palm Oil

Palm oil was physicochemically analyzed for the quality parameters including refractive index, density, acid value, iodine value, peroxide value, saponification value, and unsaponifiable matter (%) using standard methods of AOCS.

### 2.3. Experimental Procedure

Biodiesel was produced using both chemical and enzymatic transesterification of palm oil. Chemical transesterification reactions of oil were catalyzed by NaOH, KOH, and NaOCH_3_ using specified reaction conditions according to central composite response surface design (CCRD) [11, 14–16], whereas enzymatic transesterification reactions were carried out with NOVOZYME-435 and A.n. lipase as per description of Shimada et al. [17] and Shimada et al. [18].

For alkali catalyzed chemical transesterification, the reactions were carried out in a laboratory scale reactor, that is, three neck glass fabricated flask fitted with a stirrer, sampling inlet, chiller, condenser, thermometer, and heating plate [11]. Chemical transesterification reactions in each batch of oil (50 grams) were executed using specified levels of catalyst concentration, methanol to oil molar ratio, reaction temperature, reaction time according to central composite response surface design (CCRD) at fixed stirring rate, that is, 600 rpm. After transesterification, purified biodiesel was recovered from the glycerol (byproduct) by eliminating other impurities such as residual methanol and catalysts. On the other hand, enzyme catalyzed transesterification at fixed shaking speed, that is, 200 rpm, with three-step methanol addition in reaction flask, was carried out following the method as described earlier by Shimida et al.[18]. After the completion of the reaction, biodiesel was separated from the glycerol and enzyme was recovered. Biodiesel yield (%) was selected as response for the optimization studies and was described on under-study oil mass basis for transesterification reactions using the following expression:

(1) Fatty  Acid  Methyl  Esters  (FAME's)  (%Y) =Methyl  ester  produced  in  gramsGrams  of  oil  used  during  transesterification  reaction  ×100.

Furthermore, the effect of the variables, that is, catalyst concentration (*A*), reaction time (*B*), reaction temperature (*C*), and methanol : oil molar ratio (*D*) on percentage yield of biodiesel, was evaluated through CCRD. The investigated ranges for variables* A*,* B*,* C*, and* D* are described in Table 1.

A 2^4^ full-factorial CCRD design was employed during optimization using four variables with five different levels leading to 30 experiments. Each experiment was repeated three times. The data generated by 30 experiments was further statistically analyzed and used for optimization of biodiesel yield. The experimental results were analyzed by Design Expert 7 and SPSS for ANOVA, response surface plots, and diagnostics checks. The appropriate response surface models for optimization purposes based on experimental biodiesel yields were chosen for both chemical and enzymatic transesterification on the basis of ANOVA with high statistical significance, lack of fit tests, and high values of *R* ^2^. Furthermore, significance of the selected models and the individual coefficients were tested by *f* and *t*-tests [11, 15, 16].

### 2.4. Monitoring of Biodiesel Production

FTIR spectroscopic monitoring of transesterification of palm oil was executed using Interspec 200-X FTIR spectrophotometer (Spectronic Camspec Ltd., Tudor House, Barley Hill Road, Garforth, Leeds LS25 1DX, UK) equipped with mountable FTIR liquid cells. The spectra were recorded over scanning range of 500 to 5000 cm^−1^ [19, 20].

### 2.5. Compositional Analysis by GC

Fatty acids profile of the prepared palm oil biodiesel was analyzed qualitatively and quantitatively using GC/MS (Agilent Technologies 6890N) network gas chromatographic system having an inert XL mass and autoinjector. Fatty acid methyl esters of the biodiesel produced were separated using polar capillary column (100 m × 0.25 mm and film thickness 0.20 *μ*m). A 1.0 *μ*L biodiesel sample was injected into the column through split injection mode with a split ratio 1 : 100. Extra pure helium was used as carrier gas (mobile phase) with flow rate of 1.2 mL/min. Column oven temperature was programmed from 150 to 250°C @ 4°C/min while initial and final hold up times were set to be 1 and 5 min, respectively. Temperature of the injector and MS transfer line were maintained at 250 and 260°C, respectively. An electron ionization system was employed for GC/MS detection while scanning mass range varied from 30 to 550 m/z. Identification of individual fatty acids of palm oil biodiesel was executed by comprehensive comparison of their relative retention times with those of authentic standards of fatty acid methyl esters (Sigma Chemical Co., St Louis, MO, USA). For confirmation of FAMEs profile of palm oil biodiesel, MS spectra of the unknown sample were also compared with MS spectra of the same from the NIST mass spectral library provided with GC/MS system. Quantification of individual fatty acids was carried out by Agilent Technologies data handling software (Chem Station 6890) and composition was reported as relative percentage of the total peak area [11, 21].

### 2.6. Exhaust Emission Levels Analysis

Emission levels estimation was carried out using diesel engine (SD-1110) of power 20 HP and weight 210 kg equipped with a tube well situated in village nearby University of Gujrat, Gujrat, Pakistan. Palm oil biodiesel as synthesized in the current study along with its blends was employed for estimation of their emission levels. Biodiesel samples were blended with conventional fossil based petroleum diesel and different blends, that is, B5, B20, B40, B50, B80, and B100 with biodiesel percentage 5, 10, 20, 40, 50, 80, and 100%, respectively, were prepared.

### 2.7. Monitoring of Exhaust Emission Levels

Exhaust emissions monitoring from diesel engine, operated on different biodiesel blends, was carried out from the monitoring holes already provided on the diesel engine stacks. All the measurements were executed thrice when the engine was operating at their optimum load at different times to ascertain the emission behavior. For the monitoring of CO and NOx (NO + NO_2_) flue gas analyzer, that is, LANCOM-III (version V1.II, serial number 11138651 based on CTM method 034 of US EPA) fabricated with infrared and electrochemical sensor of nondispersive nature was used, whereas the estimation of particulate matter was executed using “The Casella” (particulate sampling system instrument) in compliance with ISO-9096 and BS-3405. Cellulosic filter media with pore size <10 *μ* were used in the instrument provided that it resulted in quantitative retention of PM_10_ for definite time intervals. The filter media were well assembled in a leak proof dust collecting port. The whole assembly was connected with a steal probe with the help of special tuning to withstand high temperatures associated with flue gases. After definite time interval PM retained on the surface of the filter media was measured [11].

### 2.8. Fuel Characteristics of Palm Oil Biodiesel

Fuel properties of the produced palm oil biodiesel,* namely*, density (ASTM D 5002), cetane number (ASTM D 613), pour point (ASTM D 97), cloud point (ASTM D 2500), kinematic viscosity (ASTM D 445), and ash content (ASTM D 874), and so forth, were determined using standards methods.

## 3. Results and Discussion

### 3.1. Physicochemical Characteristics of Under-Study Palm Oil

Physicochemical characteristics of palm oil used as feedstock for the production of biodiesel were evaluated which were found to be 1.454 ± 0.003, 0.88 ± 0.18, and 0.45 ± 0.04 mg KOH/g of oil, 54.60 ± 2.51 g I_2_/100 g of oil, 10.85 ± 1.17 meq/kg of oil, 198.7 ± 2.5 mg KOH/g of oil and 3.40 ± 0.02% for refractive index, density, acid value, iodine value, peroxide value, saponification value, and unsaponifiable matter (%), respectively.

### 3.2. Palm Oil Fatty Acid Methyl Esters Yields (%)

**Figure 1:**
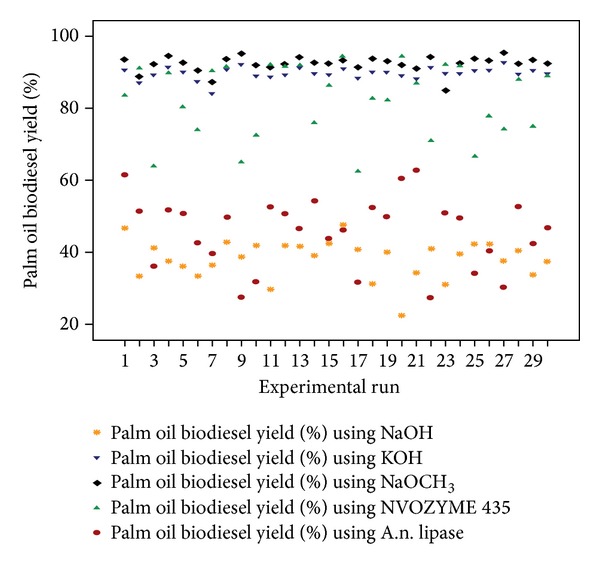
Comparative description of biodiesel yields (%) resulted from 30 experiments executed under reaction conditions defined by CCRD for chemical and enzymatic transesterification of palm oil.

Based upon experimental outputs developed from enzymatic and chemical transesterification of palm oil as per defined CCRD, comparative descriptions of biodiesel yields (%) were carried out and presented in Figure 1. Irregular trends in biodiesel yields were observed for both chemical and enzymatic transesterification reactions. The yield of biodiesel produced through chemical transesterification using NaOH, KOH, and NaOCH_3_ ranged from 22.5 to 47.4, 84.0 to 92.7, and 84.9 to 95.4%, respectively, whereas it ranged from 62.5 to 94.2 and 27.5 to 62.8% for enzymatic transesterification catalyzed by NOVOZYME-435 and A.n. lipase, respectively.

**Figure 2:**
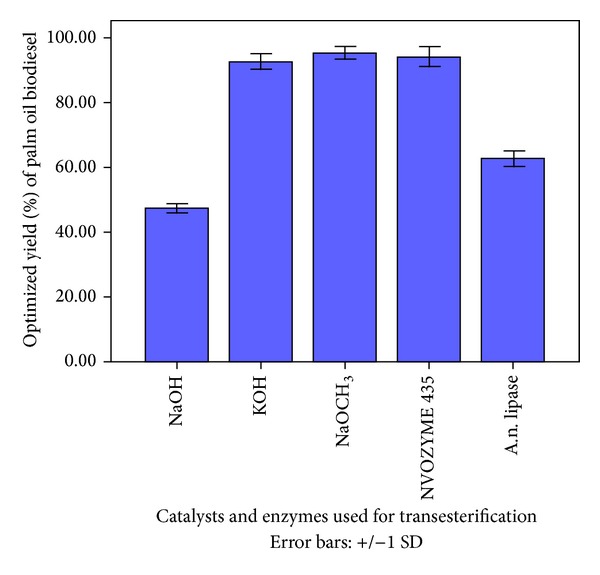
Optimized palm oil biodiesel yield (%) and ± standard deviation for chemical and enzymatic transesterification.

Based upon selected quadratic response surface design the optimized predicted biodiesel yields were used for the validation of actual experimental biodiesel yields, namely, 47.6 ± 1.5, 92.7 ± 2.5, and 95.4 ± 2.0% (Figure 2) for chemical transesterification using NaOH, KOH, and NaOCH_3_, respectively, whereas 94.2 ± 3.1 and 62.8 ± 2.4%, respectively, for NOVOZYME-435 and A.n. lipase catalyzed transesterification of palm oil (Figure 2). Experimental biodiesel yields were depicted to be well in agreement with predicted values.

### 3.3. Response Surface Quadratic Models for Optimization of Biodiesel Production

Quadratic models show that the model is best fitted for the experimental data and validated to be significant with *P* values <0.05. Nonsignificant lack of fit tests also suggested that quadratic models were best fitted for chemical transesterification of palm oil catalyzed by NaOH, KOH, and NaOCH_3_ with *P* values, that is, 0.0521, 0.7415, and 0.0813 > 0.05, respectively, as well as for enzymatic transesterification catalyzed by NOVOZYME-435 and A.n. lipase with *P* values, that is, 0.0511 and 0.2358 > 0.05, respectively. Fittness of quadratic models was also ascertained by computing *R* ^2^ and adjusted *R* ^2^ values. For NaOH, KOH, and NaOCH_3_ catalyzed transesterification of palm oil, the *R* ^2^ values were 0.9779, 0.9325, and 0.8547 and adjusted *R* ^2^ values were 0.9574, 0.8695, and 0.7191, respectively, whereas for NOVOZYME-435 and A.n. lipase catalyzed transesterification of palm oil, the *R* ^2^ values were 0.9982 and 0.9624 and adjusted *R* ^2^ values were 0.9965 and 0.9273, respectively.

### 3.4. Optimized Reaction Parameters

For NaOH catalyzed transesterification of palm oil, the highest POFAME's yield, was obtained using NaOH (0.5%) and methanol to oil molar ratio (7.5 : 1.0) at reaction temperature of 52.5°C for 75 minutes (Table 2).

When transesterification reaction using KOH as a catalyst was done, maximum POFAME's yield was achieved by conducting the reactions for 90 minutes using 0.75% KOH and 6 : 1 methanol : oil molar ratio at 45°C. Optimized reaction parameters for NaOCH_3_ catalyzed transesterification of palm oil, offering optimum POFAMEs yield, were found to be 0.75%, 6 : 1 methanol to oil molar ratio, 45°C reaction temperature, and 90 minutes reaction time. The highest POFAMEs yield was obtained for NOVOZYME-435 catalyzed transesterification of palm oil using 1.0% NOVOZYME-435 concentration, 6 : 1 methanol : oil molar ratio, 32.5°C reaction temperature, and 60 hours reaction time, whereas for A.n. Lipase catalyzed transesterification the optimal POFAMEs yield was recorded by using 1.25% A.n. lipase concentration, 9 : 1 methanol : oil molar ratio, 30°C reaction temperature, and 96 hours reaction time (Table 2).

### 3.5. FTIR Spectroscopic Monitoring of Transesterification

**Figure 3:**
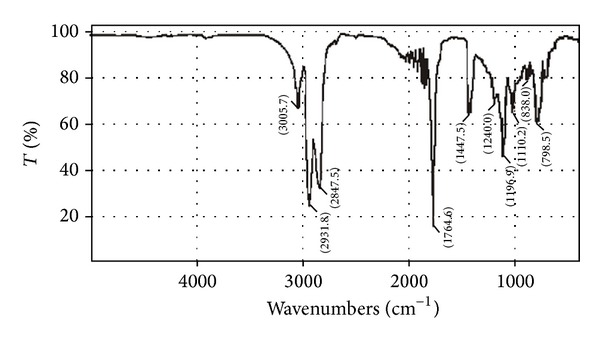
A typical FTIR spectrum of palm oil based biodiesel.

FTIR spectroscopic analysis was performed for monitoring transesterification reactions of palm oil. FTIR spectra of palm oil and palm oil derived biodiesel were recorded. The presence of IR bands in the region 1425–1447 cm^−1^ for CH_3_ asymmetric bending and 1188–1200 for O–CH_3_ stretching in all biodiesels. IR spectra clearly depicted the conversion of palm oil (i.e., triglycerides) to fatty acid methyl esters, as these IR bands were not present in the parent oil (i.e., palm oil) IR spectra as shown in Figure 3.

The region 1370–1400 cm^−1^ for O–CH_2_ groups in glycerol (moiety of TG, DG, and MG) was found to be in IR spectrum of palm oil, while in biodiesel spectrum this band was absent. Furthermore, the regions 1700–1800 cm^−1^ for C=O stretch and 2800–3000 cm^−1^ for symmetric C–H stretching were present in both the palm oil and the biodiesel IR spectra. These findings are in agreement with those of our previous studies [19, 20] as well as with some other published works [14, 22, 23].

### 3.6. ANOVA for Selected Response Surface Quadratic Models

ANOVA for response surface quadratic model (Table 3) clearly depicted that the *P* values for all three models (a, b, and c) were less than the level of significance 0.05, meaning that the quadratic models were significantly fit for the experimental results of transesterification of palm oil using three different alkaline catalysts. For model a, the main effects, that is, catalyst concentration (*A*), reaction time (*B*), and methanol to oil molar ratio (*D*), were found to be significant with *P* values lesser than 0.05, while for model b methanol : oil molar ratio (*D*) and catalyst concentration (*A*) were among the main significant effects, whereas for model c all the main effects were found to be significant.

Among all first order interaction terms, catalyst concentration × methanol to oil molar ratio (*AD*) for model a, and for model b catalyst concentration × reaction time (*AB*), catalyst concentration × reaction temperature (*AC*), catalyst concentration × methanol to oil molar ratio (*AD*), reaction time × methanol to oil molar ratio (*BD*), and reaction temperature × methanol to oil molar ratio (*CD*), whereas, for model c, catalyst concentration × reaction temperature (*AC*) and reaction time × reaction temperature (*BC*) were depicted to be significant. Among quadratic terms reaction time (*B* ^2^) and methanol to oil molar ratio (*D* ^2^) were ascertained to be significant for model a, while for model b catalyst concentration (*A* ^2^), reaction time (*B* ^2^), and methanol to oil molar ratio (*D* ^2^) were significant quadratic terms with *P* values lesser than 0.05, while for model c only methanol to oil ratio (*D* ^2^) was significant quadratic term.

On the other hand, ANOVA for quadratic models d and e and for enzymatic transesterification of palm oil catalyzed by NOVOZYME-435 and A.n. lipase, respectively, are described in Table 4. The *P* values for testing the model significance for models d and e were less than the level of significance 0.05, meaning that the quadratic models were significantly fit for the experimental results of enzyme catalyzed transesterification of palm oil. For quadratic model d, the main effects, that is, methanol to oil molar ratio (*D*) and enzyme concentration (*A*), were found to be noteworthy, while for model e the main effects, that is, enzyme concentration (*A*), reaction time (B), reaction temperature (*C*), and methanol to oil molar ratio (*D*) were found to be significant. Among all first order interaction terms, enzyme concentration × reaction time (*AB*), enzyme concentration × reaction temperature (*AC*), and enzyme concentration × methanol to oil molar ratio (*AD*) were significant for model d, whereas for model e enzyme concentration × reaction time (*AB*), enzyme concentration × reaction temperature (*AC*), and reaction time × reaction temperature (*BC*) were found to be significant first order interaction terms. Similarly, for model d, *P* values for quadric terms, that is, enzyme concentration (*A* ^2^) and reaction time (*B* ^2^), were lesser than 0.05 which ascertained their significant contribution; on the other hand, for model e among quadratic terms, reaction time (*B* ^2^), reaction temperature (*C* ^2^), and methanol to oil molar ratio (*D* ^2^) were ascertained as nonsignificant quadratic terms.

**Figure 4:**
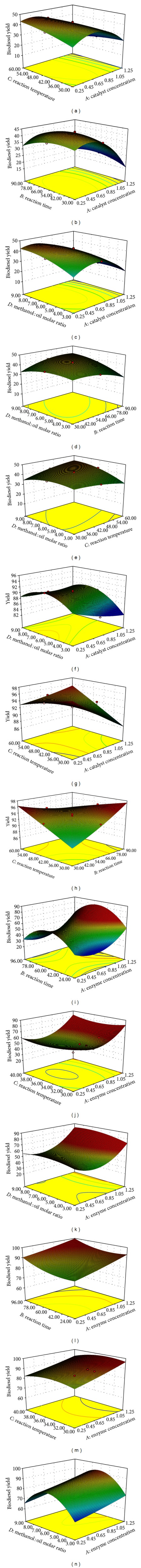
Response surface plots (a)–(h) showing significant first order interactions among different reaction parameters involved in palm oil biodiesel production via base catalyzed transesterification and response surface plots (i)–(n) showing significant first order interactions among different reaction parameters involved in palm oil biodiesel production via enzyme catalyzed transesterification.

Response surface plots (Figures 4(a)-4(n)) summarized significant response surface contributions from different reaction parameters for the optimized production of palm oil biodiesel. Response surface plots (Figures 4(a)–4(e)), showing the effect of different reaction parameters on the % yield POFAMEs, depicted that, for an increase in levels of catalyst (NaOH) concentration up to 0.5%, methanol to oil molar ratio up to 7.5 : 1, reaction time up to 75 minutes, and reaction temperature up to 52.5°C, the conversion of palm oil to palm oil fatty acid methyl esters increased and reached to maximum value, whereas beyond these levels of reaction parameters a decreasing trend in % yield of POFAMEs was observed. Impact of significant first order interaction between catalyst (KOH) concentration and methanol to oil molar ratio (Figure 4(f)) depicted highest POFAMEs while performing base catalyzed transesterification reactions of palm oil to palm oil biodiesel using 0.75% KOH concentration and 6 : 1 methanol to oil molar ratio. Higher % yield of POFAMEs was procured via KOH catalyzed transesterification of palm oil using higher KOH concentration comparative to NaOH catalyzed reactions. Similarly, impact of significant first order interactions was also ascertained by the response surface plots (Figures 4(g)–4(h)), showing the effect of reaction time and reaction temperature, catalyst concentration and reaction time on NaOCH_3_ catalyzed transesterification of palm oil for optimized production of POFAME's; the response surface plots depicted that maximum POFAME's yield was achieved when transesterification of palm oil was performed using NaOCH_3_, that is, 0.75% for a time period of 90 minutes at 45°C, whereas beyond these levels lesser POFAME's yield was observed. Response surface plots (Figures 4(i)–4(k)) depicting significant contributions of first order interactions for enzyme (A.n. lipase) catalyzed transesterification of palm oil revealed that maximum POFAMEs yield was received by performing enzyme catalyzed transesterification of palm oil using 1.25% A.n. lipase concentration and methanol to oil molar ratio 9 : 1 for a time period of 96 hours at 30.0°C, whereas above and below these levels a decrease in POFAMEs yield was observed.

On the other hand, when NOVOZYME-435 was used instead of immobilized A.n. lipase, the significant first order responses, shown in response surface plots (Figures 4(l)–4(n)), revealed that maximum POFAMEs yield was procured using NOVOZYME-435 concentration, methanol to oil molar ratio, reaction time, and reaction temperature, that is, 1.0%, 6 : 1, 60 hours, and 32.5°C, respectively. The POFAMEs yield achieved via NOVOZYME-435 catalyzed transesterification of palm oil was ascertained to be higher comparative to A.n. lipase catalyzed transesterification of palm oil.

### 3.7. Palm Oil Biodiesel Composition

Major fatty acid methyl esters profile of palm oil fatty acid methyl esters (POFAME) was revealed to consist of myristic acid methyl esters (C14:0), palmitic acid methyl esters (C16:0), palmitoleic acid methyl esters (C16:1), stearic acid methyl esters (C18:0), oleic acid methyl esters (C18:1), linoleic acid methyl esters (C18:2), and linolenic acid methyl esters (C18:3) with % compositions 1.40, 41.5, 0.20, 3.90, 38.6, 10.6 and 1.09%, respectively (Table 5). Palmitic acid methyl esters, oleic acid methyl esters, and linoleic acid methyl esters estimated in present study were comparable to those reported earlier, that is, 42.6%, 40.5%, and 10.1%, respectively, by Akoh et al. [24].

### 3.8. Exhaust Emission Profile of Biodiesel Synthesized

Pollution-free air is one of the key requirements for healthy society [25, 26]. Much epidemiological evidence has been provided by various researchers revealing direct association between health effects and toxic exhaust emission levels. In this regard, particulate matters, NOx, CO, and SO_2_ are considered as the main factors of concern. Reductions in pulmonary functions, asthma, lung function decrements, and premature deaths, and so forth are reported to be associated with these pollutants. Furthermore, high affinity of CO for hemoglobin to form carboxyhaemoglobin is assumed as main attributor of CO toxicity causing acute poisoning leading to death [24, 27–31]. Emissions resulted from fuel burning have gained importance owning to their adverse health impacts. Currently much attention is focused throughout the world to minimize the levels of these emissions by developing alternative environment friendly fuels. During current research work biodiesel was synthesized and then subjected to exhaust emission level studies.

**Figure 5:**
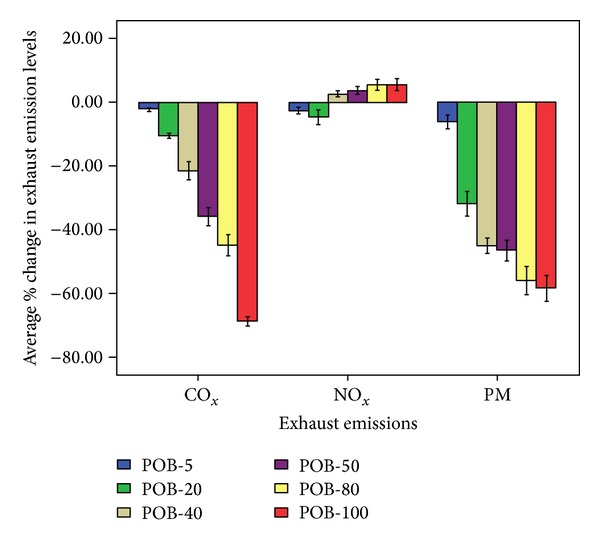
% change in exhaust emissions (CO, NOx, and PM) from engine exhaust operated on different palm oil biodiesel blends comparative to engine exhaust operated on conventional petrodiesel.

Figure 5 showed considerable decrease in engine exhaust emissions profile, that is, CO and PM matter from palm oil based biodiesel, and its blends when compared with petrodiesel. On the average basis % change in CO emission levels from engine exhaust operating on POB-5, POB-20, POB-40, POB-50, POB-80, and POB-100 was found to be −2.1 ± 0.3, −10.5 ± 0.7, −21.5 ± 2.7, −35.9 ± 2.7, −44.8 ± 3.3, and −68.7 ± 1.4%, respectively, whereas % change in particulate matter (PM) emissions was revealed to be −6.2 ± 2.1, −31.8 ± 3.9, −44.9 ± 2.3, −46.5 ± 3.2, −55.9 ± 4.5, and −58.4 ± 4.0%, respectively (Figure 5), comparative to conventional petrodiesel. On the other hand, an irregular trend in NOx emissions was depicted; NOx emissions from engine exhaust operated on POB-40, POB-50, POB-80, and POB-100 were found to be higher than engine exhaust emissions operated on conventional petrodiesel with % change, that is, 2.6 ± 0.9, 3.7 ± 1.2, 5.4 ± 1.7, and 5.5 ± 1.8%, respectively, whereas in case of POB-5 and POB-20 NOx emissions were found to be lesser than conventional petrodiesel with % changes −2.6 ± 1.0 and −4.6 ± 2.3%, respectively. Graboski and McCormick [9] reported 12% increase in NOx emission levels using 100% pure soy biodiesel comparative to the petroleum diesel [9], whereas 20% blend of pure soy biodiesel in petrodiesel depicted only 2 to 4% rise in NOx emission levels comparative to conventional diesel. The increased levels of NOx emissions even at small scale can negatively impact the biodiesel use [9].

### 3.9. Fuel Properties of Palm Oil Biodiesel

The fuel characteristics, that is, kinematic viscosity ((mm^−2^/s) 40°C), ash content (%), cloud point (°C), pour point (°C), higher heating value (MJ/Kg), and cetane number for POFAME, were depicted to be 4.31 ± 0.23 mm^−2^/s, 0.032 ± 0.025%, 11.8 ± 1.5°C, 7.49 ± 1.20°C, 42.66 ± 0.54 MJ/Kg, and 52.44 ± 2.29, respectively. Benjamin et al. [32] reported the fuel properties, namely, 4.71 mm^−2^/s, 16.0°C, and 50.0 for kinematic viscosity, cloud point, and cetane number of palm oil biodiesel, respectively. The results presented by Benjumea et al. [33] are comparable to the findings of current research.

## 4. Conclusions

Results ascertained the optimized production of palm oil based biodiesel using response surface methodology. The optimal levels of palm oil biodiesel via base catalyzed transesterification was 95.4 ± 2.0% using NaOCH_3_, while for enzyme catalyzed transesterification the maximum biodiesel yield procured was 94.2 ± 3.1% using NOVOZYME-435. NaOCH_3_ was therefore proved to be most effective catalyst in the present study among other alkaline catalysts (NaOH, KOH, and NaOCH_3_) employed, whereas NOVOZYME-435 was the most effective lipase catalyst. Furthermore, synthesized biodiesel was found to be green fuel in terms of considerable reduction in exhaust emissions comparative to conventional petrodiesel and it showed fuel properties technically in compliance with ASTM D6751 and EN 14214 standards.

## Figures and Tables

**Table 1:** CCRD design summary of different levels of reaction variables used for transesterification of palm oil for chemical and enzymatic transesterification.

Factor	Name	Units	Low level	High level
*A* ^a^	Catalyst concentration	%	00.25	01.25
*A* ^b^	Enzyme concentration	%	00.25	01.25
*B* ^a^	Reaction time	Minutes	30.00	90.00
*B* ^b^	Reaction time	Hours	24.00	96.00
*C* ^a^	Reaction temperature	°C	30.00	60.00
*C* ^b^	Reaction temperature	°C	30.00	35.00
*D* ^a&b^	Alcohol : oil molar ratio		03.00	09.00

^a^CCRD design for chemical transesterification of palm oil.

^b^CCRD design for enzymatic transesterification of palm oil.

**Table 2:** Optimized reaction parameters for biodiesel production using chemical and enzymatic transesterification of under-study feedstock.

Catalyst/enzyme	C/E concentration	Reaction time	Reaction temperature	Methanol : oil molar ratio	Biodiesel yield
NaOH	0.5%	75 Min	52.5°C	7.5 : 1	47.6%
KOH	0.75%	90 Min	45.0°C	6 : 1	92.7%
NaOCH_3_	0.75%	90 Min	45.0°C	6 : 1	95.4%
NOVOZYME-435	1.0%	60 Hrs	32.5°C	6 : 1	94.2%
A.n.Lipase	1.25%	96 Hrs	30.0°C	9 : 1	62.8%

**Table 3:** Response surface quadratic model analysis of variance (ANOVA) table for chemical transesterification of palm oil.

Source	df	SS (MS)^a^	SS (MS)^b^	SS (MS)^c^	*F* value (*P* value)^a^	*F* value (*P* value)^b^	*F* value (*P* value)^c^
Model	14	76.24 (5.45)	799.32 (57.09)	118.98 (8.50)	14.80 (<0.0001)	47.51 (<0.0001)	6.30 (0.0005)
*A*-catalyst concentration	1	*12.54* (*12.54*)	*436.91* (*436.91*)	*27.05* (*27.05*)	*34.09* (<*0.0001*)	*363.60* (<*0.0001*)	*20.06 *(*0.0004*)
*B*-reaction time	1	*7.72* (*7.72*)	*0.74* (*0.74*)	*15.33* (*15.33*)	*20.98* (*0.0004*)	*0.61* (*0.4463*)	*11.37 *(*0.0042*)
*C*-reaction temperature	1	*0.77* (*0.77*)	*3.23* (*3.23*)	*7.17* (*7.17*)	*2.08* (*0.1694*)	*2.69* (*0.1221*)	*5.32* (*0.0358*)
*D*-alcohol : oil molar ratio	1	*19.21* (*19.21*)	*32.67* (*32.67*)	*7.66* (*7.66*)	*52.21* (<*0.0001*)*1*)	*27.19* (*0.0001*)	*5.68 *(*0.0308*)
*AB*	1	*0.092* (*0.092*)	*7.02* (*7.02*)	*2.86* (*2.86*)	*0.25* (*0.6252*)	*5.84* (*0.0288*)	*2.12 *(*0.1662*)
*AC*	1	*0.42* (*0.42*)	*22.09* (*22.09*)	*11.53* (*11.53*)	*1.14* (*0.3026*)	*18.38* (*0.0006*)	*8.55* (*0.0105*)
*AD*	1	*2.17* (*2.17*)	*17.64* (*17.64*)	*0.0025* (*0.0025*)	*5.89* (*0.0282*)	*14.68* (*0.0016*)	*0.001854 *(*0.9662*)
*BC*	1	*0.66* (*0.66*)	*2.10* (*2.10*)	*11.39* (*11.39*)	*1.79* (*0.2003*)	*1.75* (*0.2057*)	*8.45* (*0.0109*)
*BD*	1	*0.47* (*0.47*)	*23.52* (*23.52*)	*0.41* (*0.41*)	*1.28* (*0.2748*)	*19.58* (*0.0005*)	*0.30 (0.5896*)
*CD*	1	*0.21* (*0.21*)	*18.49* (*18.49*)	*5.00* (*5.00*)	*0.58* (*0.4576*)	*15.39* (*0.0014*)	*3.70* (*0.0734*)
*A* ^2^	1	*0.80* (*0.80*)	*222.79* (*222.79*)	*0.11* (*0.11*)	*2.16* (*0.1621*)	*185.41* (<*0.0001*)	*0.081* (*0.7805*)
*B* ^2^	1	*2.81* (*2.81*)	*23.47* (*23.47*)	*0.69* (*0.69*)	*7.65* (*0.0144*)	*19.53* (*0.0005*)	*0.52* (*0.4839*)
*C* ^2^	1	*0.58* (*0.58*)	*3.86* (*3.86*)	*0.015* (*0.015*)	*1.57* (*0.2288*)	*3.21* (*0.0934*)	*0.011* (*0.9176*)
*D* ^2^	1	*23.45* (*23.45*)	*19.82* (*19.82*)	*27.13* (*27.13*)	*63.75* (<*0.0001*)	*16.49* (*0.0010*)	*20.12* (*0.0004*)
Residual	15	5.52 (0.37)	18.02 (1.20)	20.23 (1.35)			
Lack of fit	10	*3.11* (*0.31*)	*16.27* (*1.63*)	*17.81* (*1.78*)	*0.64* (*0.7415*)	*4.64* (*0.0521*)	*3.69* (*0.0813*)
Pure error	5	*2.41* (*0.48*)	*1.75* (*0.35*)	*2.42* (*0.48*)			

Cor total	29	81.76	817.35	139.20			

SS (MS) = sum of squares (mean square).

Model a represents quadratic model based on experimental results of KOH catalyzed transestrification of under-study feedstock.

Model b represents quadratic model based on experimental results of NaOH catalyzed transestrification of under-study feedstock.

Model c represents quadratic model based on experimental results of NaOCH_3_ catalyzed transestrification of under-study feedstock.

**Table 4:** Response surface quadratic model analysis of variance (ANOVA) for enzymatic transesterification of palm oil.

Source	df	SS (MS)^d^	SS (MS)^e^	*F* value (*P* value)^d^	*F* value (*P* value)^e^
Model	14	2666.17 (190.44)	2923.96 (208.85)	27.40 (<0.0001)	588.71 (<0.0001)
*A*-enzyme concentration	1	*1093.29* (*1093.29*)	*226.00* (*226.00*)	*157.33* (<*0.0001*)	*637.04* (<*0.0001*)
*B*-reaction time	1	*28.38* (*28.38*)	*120.55* (*120.55`*)	*4.08* (*0.0615*)	*339.79* (<*0.0001*)
*C*-reaction temperature	1	*4.07* (*4.07*)	*2.69* (*2.69*)	*0.59* (*0.4558*)	*7.59* (*0.0148*)
*D*-alcohol : oil molar ratio	1	*63.73* (*63.73*)	*12.62 (12.62)*	*9.17* (*0.0085*)	*35.56* (*<0.0001*)
*AB*	1	*89.78* (*89.78*)	*7.56* (*7.56*)	*12.92* (*0.0027*)	*21.32* (*0.0003*)
*AC*	1	*60.45* (*60.45*)	*6.50* (*6.50*)	*8.70* (*0.0099*)	*18.33* (*0.0007*)
*AD*	1	*77.00* (*77.00*)	*0.42* (*0.42*)	*11.08* (*0.0046*)	*1.19* (*0.2924*)
*BC*	1	*13.51* (*13.51*)	*1.69* (*1.69*)	*1.94* (*0.1836*)	*4.76* (*0.0454*)
*BD*	1	*24.26* (*24.26*)	*0.16* (*0.16*)	*3.49* (*0.0814*)	*0.45* (*0.5121*)
*CD*	1	*3.52* (*3.52*)	*0.49* (*0.49*)	*0.51* (*0.4878*)	*1.38* (*0.2582*)
*A* ^2^	1	*49.60* (*49.60*)	*0.055* (*0.055*)	*7.14* (*0.0174*)	*0.16* (*0.6993*)
*B* ^2^	1	*110.22* (*110.22*)	*2.13* (*2.13*)	*15.86* (*0.0012*)	*5.99* (*0.0271*)
*C* ^2^	1	*5.34* (*5.34*)	*1.95* (*1.95*)	*0.77* (*0.3945*)	*5.50* (*0.0331*)
*D* ^2^	1	*3.73* (*3.73*)	*52.88* (*52.88*)	*0.54* (*0.4750*)	*149.05* (<*0.0001*)
Residual	15	104.24 (6.95)	5.32 (0.35)		
Lack of fit	10	*83.11* (*8.31*)	*4.81* (*0.48*)	*1.97* (*0.2358*)	*4.68* (*0.0511*)
Pure error	5	*21.13* (*4.23*)	*0.51* (*0.10*)		

Cor total	29	2770.41	2929.28		

SS (MS) = sum of squares (mean square).

Model d = represents quadratic model based on experimental results of A.n. lipase catalyzed transestrification of under-study feedstock.

Model e = represents quadratic model based on experimental results of NOVOZYME-435 catalyzed transestrification of under-study feedstock.

**Table 5:** Major fatty acid methyl esters of palm oil biodiesel.

Sr. no.	Fatty acid methyl ester	Retention times	POFAME's
1	Myristic acid (C14:0)	12.0920	1.40 ± 0.11
2	Palmitic acid (C16:0)	14.5991	41.50 ± 2.18
3	Palmitoleic acid (C16:1)	—	0.20 ± 0.01
4	Stearic acid (C18:0)	17.8101	3.90 ± 0.14
5	Oleic acid (C18:1)	18.896	38.6 ± 1.89
6	Linoleic acid (C18:2)	20.3148	10.6 ± 1.03
7	Linolenic acid (C18:3)	22.0776	1.09 ± 0.10
8	Arachidic acid (20:0)	23.4130	0.03 ± 0.01
9	Erucic acid (C22:1)	25.9340	—

## Abbreviations

RSM: Response surface methodology
CCRD: Central composite response surface design
NOVOZYME-435: Lipase acrylic resin from Candida Antarctica
A.n. Lipase: Lipase from* Aspergillus niger*
FTIR Spectroscopy: Fourier transform infrared spectroscopy
GC-MS: Gas chromatograph equipped with mass spectrometric detector
*A*: Catalyst/enzyme concentration
*B*: Reaction time
*C*: Reaction temperature
*D*: Methanol : oil molar ratio.
